# Effect of Zr Addition on the Microstructure and Multi-Environment Tribological Behavior of MoS_2_-Zr Composite Films

**DOI:** 10.3390/nano16050299

**Published:** 2026-02-26

**Authors:** Qingye Wang, Shuang Liang, Jicheng Ding, Zhengxuan Lu, Dongcai Zhao, Xingguang Liu, Jun Zheng

**Affiliations:** 1 School of Materials Science and Engineering, Anhui University of Technology, Maanshan 243002, China; wangqingye585156@gmail.com (Q.W.); liangshuang0106@126.com (S.L.); luzhengxuan2001@outlook.com (Z.L.); zhaodongc@163.com (D.Z.); sdwfcllxg@126.com (X.L.); 2 China International Science and Technology Cooperation Base on Intelligent Equipment Manufacturing in Special Service Environment, Anhui University of Technology, Maanshan 243002, China; 3 Key Laboratory of Green Fabrication and Surface Technology of Advanced Metal Materials, Ministry of Education, Anhui University of Technology, Maanshan 243002, China

**Keywords:** MoS_2_ film, Zr doping, microstructure, multi-environment, tribology

## Abstract

Molybdenum disulfide (MoS_2_) films are promising solid lubricants for aerospace and other advanced applications, yet their tribological performance is highly sensitive to environmental conditions. To enhance environmental adaptability, Zr-doped MoS_2_ composite films were prepared by magnetron co-sputtering, and their composition, microstructure, mechanical properties, and tribological behavior were systematically investigated. The results showed that the as-deposited MoS_2_ films exhibited a nearly stoichiometric sulfur-to-molybdenum ratio (S/Mo ≈ 2), while the Zr-doped MoS_2_ composite films showed sulfur-deficient, sub-stoichiometric ratios (S/Mo < 2). Pure MoS_2_ films displayed a porous columnar structure, whereas with the incorporation of Zr, the columnar structure becomes progressively more compact. Moreover, the film structure transitions from a purely crystalline form to a two-phase structure with both crystalline and amorphous phases coexisting. The hardness and elastic modulus of the films increased with the addition of Zr, mainly due to the densification of the structure and the disorder introduced in the film. Moderate Zr doping markedly improved the friction and wear performance of composite films across vacuum, atmospheric, and humid environments. The optimal film achieved a coefficient of friction (COF) of 0.02 and wear rate of 6.23 × 10^−8^ mm^3^/N·m in vacuum and COFs of 0.10 with low wear rates in both atmospheric and humid conditions. By adjusting the Zr target power to modulate Zr content, the crystallographic orientation and microstructure of MoS_2_-Zr composite films could be tailored, thereby regulating their mechanical and tribological properties. This study provides theoretical guidance for the application of metal-doped MoS_2_ composite films under alternating environmental conditions.

## 1. Introduction

In the field of advanced aerospace technology such as deep-space exploration and on-orbit servicing, the reliability of moving mechanisms (e.g., solar array deployment mechanisms and scanning pointing mechanisms) directly determines the success or failure of a mission for spacecraft, valued at hundreds of millions of dollars. These precision mechanical systems must endure tens of thousands of fault-free cycles under extreme conditions, including ultra-high vacuum (10^−3^ Pa), thermal cycling between −150 °C and 150 °C, and atomic oxygen irradiation [1,2,3]. Such demanding conditions impose nearly stringent requirements on solid lubricating materials: they must maintain ultra-low friction coefficients in vacuum while resisting performance degradation when accidentally exposed to atmospheric or high-humidity environments [4,5]. Molybdenum disulfide (MoS_2_), as a typical layered solid lubricant, exhibits an ultra-low friction coefficient in vacuum due to its unique S-Mo-S sandwich structure, where interlayer shear occurs via weak van der Waals forces [6,7,8]. This outstanding lubricating property has established MoS_2_ as the “space lubricant” and made it widely used in the moving mechanisms of various spacecraft and launch vehicles [9,10]. However, escalating mission complexity (e.g., lunar space stations, Mars sample return) intensifies multi-environmental coupling challenges at lubricant interfaces. For instance, during atmospheric entry or cabin depressurization, lubricant films may undergo abrupt transitions between vacuum, atmospheric, and high-humidity conditions within hours [11]. Meanwhile, the films are typically required to exhibit a moderate surface roughness to maintain stable friction and avoid localized adhesion or delamination. The pure MoS_2_ film, characterized by their loose columnar structure, exhibits critical vulnerabilities: its open lamellar structure readily adsorbs water molecules to form intercalation compounds, causing friction coefficients to surge above 0.2, while the wear rate increases by two to three orders of magnitude. Moreover, the exposure of active edge sites due to the porous columnar crystalline structure accelerates oxidative corrosion, further exacerbating the risk of lubrication failure [12,13]. Consequently, enhancing the multi-environmental adaptability of MoS_2_ films is imperative.

To address these challenges, extensive research has been conducted, and doping elements (Ti, Al, Ag, Ni, C and O, etc.) has been proven to be an effective strategy for significantly improving the comprehensive properties of MoS_2_ films [7,14,15,16]. For example, Hu et al. [7] have studied the effects of Ti content on the tribological and corrosion performances of MoS_2_-Ti films. The results showed that the MoS_2_-Ti composite film possessed the lowest average friction coefficient of (COF) 0.049 at 13.89 at. % Ti content. While the best wear resistance and corrosion resistance of film was achieved at 15.17 at. % Ti content. These were ascribed to the denser structure and good mechanical properties by doping proper Ti content in films. Li et al. [17] have deposited the MoS_2_/Zr coatings and investigated the tribological and corrosive properties in high-humidity conditions. With the addition of Zr, the microstructure of coating changes gradually from the nano-multilayer to an amorphous structure. Due to the formation of a special nano-structure, the film could maintain the relative low COF of 0.05 in 78% RH humidity. Meanwhile, the corrosion resistance of MoS_2_/Zr coating was also improved, ascribed to the formed Zr oxide and dense nano multilayer structure. In Ye et al.’s work [18], both the maximum hardness and adhesion were achieved in the MoS_2_ + Zr composite coatings by appropriately adjusting the Zr doping content. Jin et al. [15] have explored the tribological properties of MoS_2_ film by doping softer metal Ag. Results revealed that the Ag-MoS_2_ film was more suitable for applying in high sliding speed areas. The metal dopants changed the structure of the MoS_2_ film and increased the hardness of the film, thereby significantly improving the tribological performance. On the other hand, Ju et al. [19] have investigated the properties of MoS_2_ films by doping nitrogen. They found that all Mo-S-N films exhibited higher hardness and oxidation resistance than that of MoS_2_ films due to the formation of Mo-N bonds. The Mo-S-N films showed excellent tribological properties ranging from room temperature to 400 °C. In Bondarev et al.’s [16] work, Mo-S-O films were fabricated in the Ar + O atmosphere with various proportions of oxygen. The composite film showed a decreased COF of 0.02, lower than the pure MoS_2_ film. The ultra-thin crystalline MoS_2_ tribolayer induced by the incorporated oxygen could be responsible for the low COF.

Although researchers have studied the influence of different doping elements on the performance of MoS_2_ films, the tribological properties of films are mostly studied in a single environment, and there is a lack of research on the tribological properties in an alternating environment. Thus, in this work, the MoS_2_-Zr composite films with different Zr contents were prepared by adjusting the power of Zr target power. The microstructure, mechanical, and tribological properties of MoS_2_-Zr films were systematically investigated; especially, the tribological property various environments, such as in air, vacuum, and humidity, were extensively explored. This study will contribute to the development and application of MoS_2_-based film in complex working conditions.

## 2. Experimental

### 2.1. Films Deposition

The MoS_2_-Zr composite thin films in this experiment were fabricated using a laboratory-scale magnetron sputtering deposition system equipped with three circular targets. Three pulsed DC power supplies were connected to the high-purity MoS_2_ target (99.9% purity, Φ 97 mm × 4 mm) and Zr target (99.7% purity, Φ 101.6 mm × 6 mm), respectively, operating at a pulse frequency of 40 kHz and a duty cycle of 70%. We selected 316 L stainless steel discs (Φ 26 mm × 3 mm; for friction and adhesion tests) and single-crystal silicon (for SEM, XRD, XPS, TEM, and hardness tests) wafers as substrates. To enhance adhesion and minimize substrate surface effects, the substrates underwent the following pretreatment protocol: (1) Single-crystal silicon wafers were single-side polished, while stainless steel substrates were sequentially ground with 1200-grit abrasive paper and mirror-polished using 2.5 μm diamond paste with nylon polishing cloth; (2) The polished stainless steel substrates underwent ultrasonic cleaning in acetone (20 min), anhydrous ethanol (20 min), and deionized water (10 min), followed by drying with compressed air. Pretreated substrates were mounted on a rotating holder (3 rpm) in the deposition chamber, which maintained a base pressure of 5 × 10^−3^ Pa with high-purity argon (99.999%) as the working gas. Prior to deposition, the substrates were subjected to −1000 V bias glow discharge cleaning for 20 min to remove surface contaminants and activate the substrate surface, followed by 5 min of target pre-sputtering. The deposition process comprised two stages: initial deposition of a Zr interlayer to improve the adhesion force between the substrate and films, followed by co-deposition of MoS_2_-Zr composite films with varying Zr target power (0–300 W) for 90 min. The resultant films were designated as Z1–Z5, corresponding to Zr target powers of 0, 50, 100, 200, and 300 W, respectively. Detailed deposition parameters are summarized in Table 1.

### 2.2. Films Characterization

The surface and cross-sectional morphologies of the films were characterized by field emission scanning electron microscopy (SEM, TESCAN MIRA3, Bron, Czechia) in secondary electron (SE) mode, coupled with energy-dispersive X-ray spectroscopy (EDS) for chemical composition analysis. EDS analysis was employed for semi-quantitative compositional comparison under identical deposition conditions. X-ray photoelectron spectroscopy (XPS, Thermo ESCALAB 250, Waltham, MA, USA) with Al Kα radiation (hν = 1486.6 eV) at 15 kV and 15 mA was employed to investigate the chemical states and bonding configurations of elements. The base pressure was better than 1 × 10^−8^ Pa, and the spot size of the analyzed area was 0.196 mm^2^ (spot diameter: 400 μm). Phase structures were analyzed using X-ray diffraction (XRD, Ultima IV, Rigaku, Tokyo, Japan) with Cu Kα radiation, and the range of scanning is 10–60° with a step size of 0.02°. For further investigating the microstructure of the film, transmission electron microscopy (TEM, FEI Talos F200X, Waltham, MA, USA) with a field emission gun operating at 200 kV was adopted and a focused ion beam system was used to prepare the TEM cross-sectional sample. Nanoindentation (MTS G200, MTS, Eden Prairie, MN, USA) was conducted using a calibrated diamond Berkovich tip (area function and frame stiffness calibrated on fused silica). Tests were performed at 2 mN with a 30–10–30 s load/hold/unload cycle, and the hardness/modulus were extracted via the Oliver–Pharr method using a Poisson’s ratio of 0.25. At least five indents were made per sample, with depths < 10% of the film thickness. Rockwell C indentation tests were carried out using a diamond cone indenter (120° cone angle, 200 μm tip radius) under a load of 60 kgf to provide a qualitative comparison of coating–substrate adhesion. Owing to the non-hardened 316 L substrate, the results are discussed in a comparative rather than standardized (VDI 3198) framework. Tribological properties were assessed using an Antonpaar TRB^3^ ball-on-disc tribometer (Anton Paar, Graz, Austria) with a 6.35 mm diameter 440 C steel ball as the counterpart. Standard test parameters included a 2 N normal load and 5 mm wear track radius. The tribological tests were conducted under three environmental conditions: ambient atmosphere (500 rpm, 30 min), vacuum at 8 × 10^−3^ Pa (1000 rpm, 60 min), and a humid environment with 85–95% relative humidity (500 rpm, 30 min). Wear volume was calculated by measuring the cross-sectional area of wear tracks using a profilometer (KLA-Tencor Alphastep P-7, KLA, Milpitas, CA, USA). Wear rate (K) was derived from K = V/S·F, where V (mm^3^) is the volumetric loss, S (m) is the sliding distance, and F (N) is the applied load. For each condition and each sample, at least three independent tests were performed to ensure repeatability, and the reported friction coefficients and wear rates represent average values. The worn surfaces were further analyzed by optical microscopy on both the film and counterpart ball.

## 3. Results and Discussion

The element compositions of MoS_2_-Zr films deposited with various Zr target powers are illustrated in Table 2. The total atomic percentage of elements in films were normalized to 100 at. %. With the increase in Zr target power, the Zr content was linearly increased from 3.60 at. % to 19.03 at. %. This implies the Zr content can precisely adjust by changing the power parameter [17,18]. The Mo and S contents in films were decreased simultaneously, while the S decreased even faster than Mo. The S/Mo atomic ratios (1.9 to 1.8) were lower than that of the standard MoS_2_ target (S/Mo = 2) in all deposited MoS_2_-Zr composite films. Sub-stoichiometric sulfur in MoS_2_-based composite films has also been reported previously by other authors [17,19]. The sulfur loss could be attributed to two primary reasons: Firstly, the lighter sulfur atoms are more susceptible to being sputtered away under the re-sputtering effect, where high-energy particles impinging on the film surface preferentially remove lighter elements. Secondly, ionized sulfur species tend to chemically react with residual oxygen, nitrogen, or hydrogen present in the vacuum chamber environment. These resultant compounds are subsequently evacuated through the pumping system, thereby leading to progressive sulfur depletion in the deposited film [20,21].

**Figure 1:**
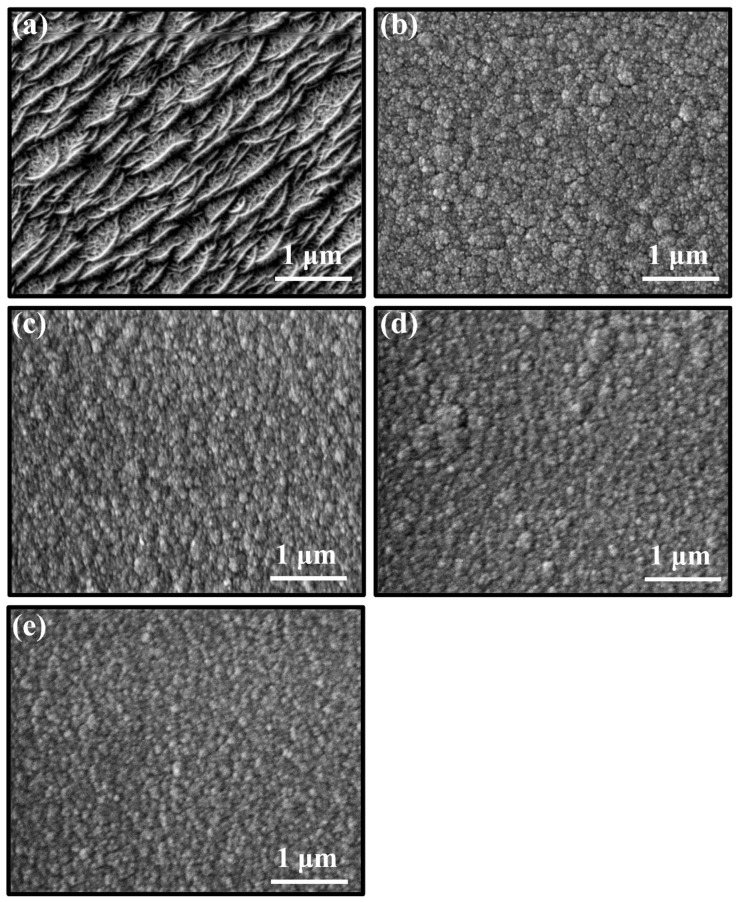
Surface morphologies of MoS_2_-Zr composite films with various Zr contents: (**a**) Z1; (**b**) Z2; (**c**) Z3; (**d**) Z4; and (**e**) Z5 film.

**Figure 2:**
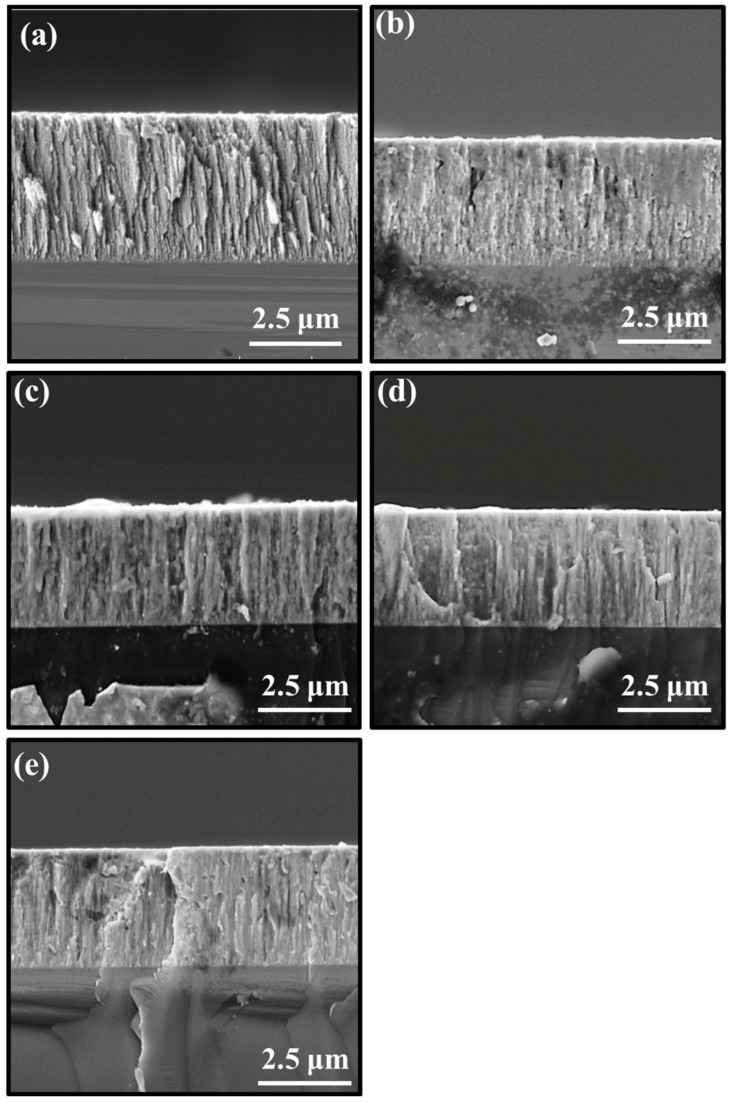
Cross-sectional morphologies of MoS_2_-Zr composite films with various Zr contents: (**a**) Z1; (**b**) Z2; (**c**) Z3; (**d**) Z4; and (**e**) Z5 film.

Surface morphologies of the MoS_2_-Zr composite films deposited at different Zr target powers are presented in Figure 1. It can be seen that the surface of the pure MoS_2_ film showed typically worm-like morphology due to low deposition temperature and shading effect during the growth, and these “worms” uniformly distributed on the surface with obvious gaps [16,22]. For the Z2 film, deposited at low Zr target power, the surface presented a cauliflower-like structure composed of aggregated particles. With a further increase in the target power, as shown in Figure 1c–e, a similar “island-like” appearance was found, indicating that the film was the island-like growth. The size of particles on the surface of films became smaller [23,24]. Meanwhile, the surface particles became compact and fewer micro-pores/defects could be observed in the boundary of the particles. The cross-sectional morphologies of films are displayed in Figure 2. For the MoS_2_ film without Zr doping, the typical loose and columnar structure is observed in Figure 2a. The cross-sectional images became denser and the loose columnar structure gradually weakened with increasing Zr target power [25]. Moreover, the thickness of films decreased from 3.8 μm to 2.6 μm with an increase in target power from 0 to 300 W. These results suggest that Zr doping promotes the densification of the film. Upon incorporation of metallic Zr, its strong thermodynamic affinity for oxygen may lead to preferential bonding between residual oxygen and Zr, as evidenced by the formation of zirconium oxide species observed in the XPS analysis part. Rather than implying a direct reduction in total oxygen content, this behavior suggests that oxygen can be chemically stabilized in the form of Zr-O bonds. Such stabilization may reduce the interaction of oxygen with Mo-S bonds and modify the activity of growth sites on the MoS_2_ surface, particularly the edge facets (100) and (110). The possible passivation of these active sites can suppress the development of a loose columnar morphology, thereby reducing grain size, minimizing intergranular voids, and promoting a denser film microstructure [26,27].

**Figure 3:**
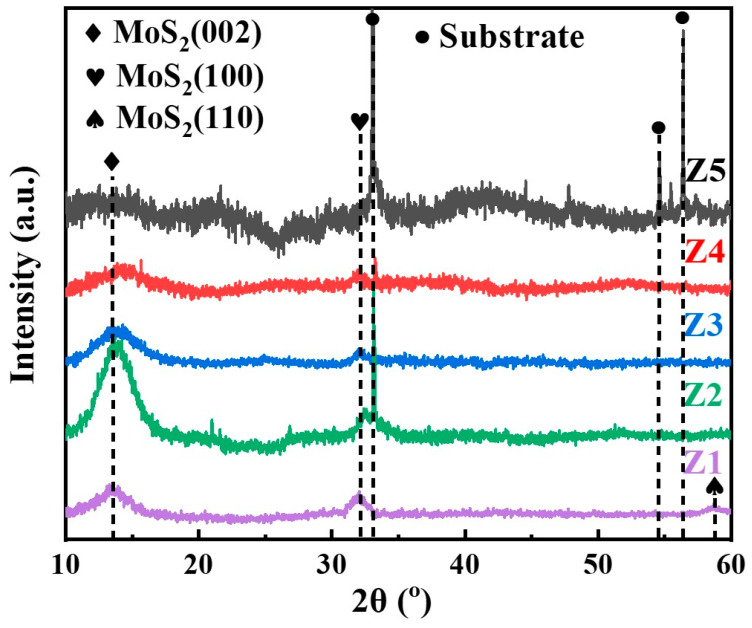
XRD patterns of MoS_2_-Zr composite films with various Zr contents.

The XRD patterns of all MoS_2_-Zr composite films are shown in Figure 3. The pattern for the MoS_2_ (Z1) film primarily features three peaks according to JCPDS card (PDF# 37-1492) at approximately 2θ = 13°, 33°, and 59°, corresponding to the (002), (100), and (110) crystal planes of MoS_2_, respectively [26,28]. This film is classified as a randomly oriented film, exhibiting a porous columnar structure, as confirmed by the SEM image in Figure 2a. For the composite film deposited at 50 W Zr target power, the (002) peak is the strongest peak. The incorporation of Zr induces a rearrangement of crystal nuclei and promotes the growth of (002) crystal planes parallel to the substrate, which is also beneficial for reducing the friction coefficient [29]. Furthermore, Lauwerens et al. [30] demonstrated that the (002) preferred orientation tends to form under low S/Mo ratios. As shown previously in Table 1, the S/Mo ratio exhibits a decreasing trend with increasing Zr content. With further increase in the Zr target power, the characteristic peaks of MoS_2_ (100) and (110) peaks almost disappeared, and there was only a broad and low (002) peak, which means the film transformed to an amorphous structural characteristic. The incorporation of metal elements into MoS_2_ films typically leads to reduced crystallinity, as also reported in previous studies [14,16]. For instance, Li et al. [17] found that the (002) peak of MoS_2_-Zr coating disappeared and the coating appeared amorphous with the increase in Zr content. A similar result was also observed in Shi et al.’s [31] work, where the (002) peak of MoS_2_-Ti film became weaker and broader, indicating a phase transformation from a columnar crystalline to an amorphous structure. These phenomena may be attributed to the lattice distortion induced by the incorporation of metals.

**Figure 4:**
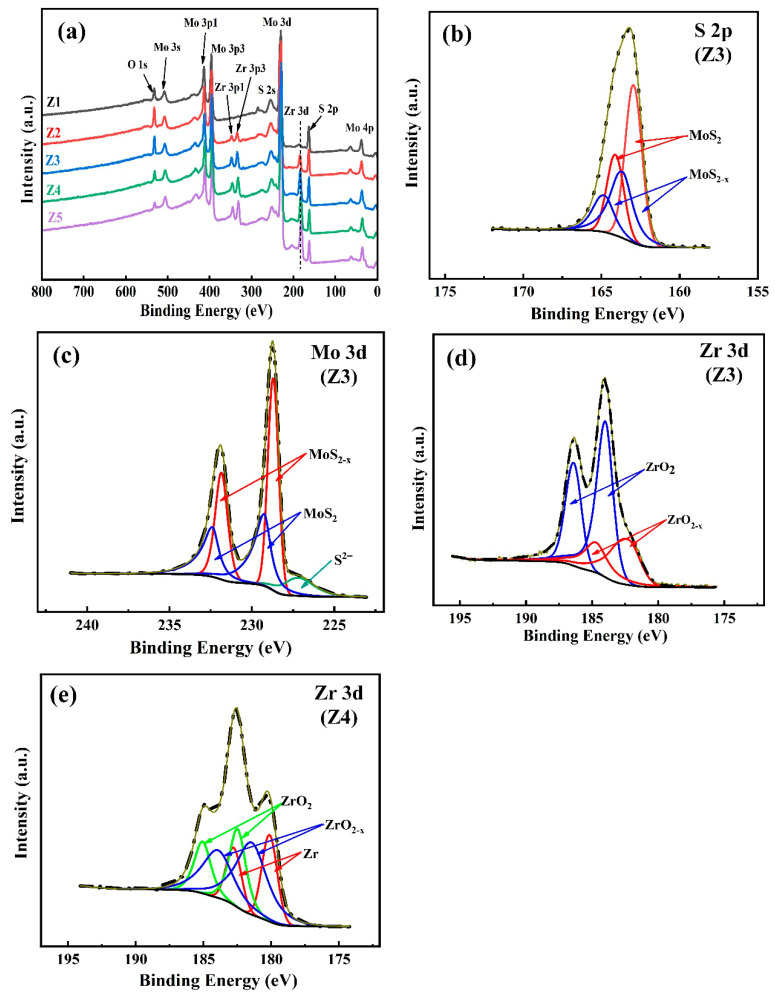
Typical XPS spectra of the MoS_2_-Zr composite films: (**a**) overall spectra, (**b**) decomposition of S 2p spectrum for Z3 film, (**c**) decomposition of Mo 3d spectrum for Z3 film, (**d**) decomposition of Zr 3d spectrum for Z3 film, and (**e**) decomposition of Zr 3d spectrum for Z4 film.

The chemical bonds of MoS_2_-Zr composite films were characterized by the XPS technique. The XPS survey spectrums of MoS_2_-Zr composite films with different target powers are shown in Figure 4. It can be seen that the composite film is mainly composed of Mo, S, and Zr in Figure 4a. All the measured binding energies were calibrated by using the work function (Φ_SA_) method [32,33]. Since the sum of C 1 s binding energy and Φ_SA_ is constant at 289.58 eV, the C 1 s peak can be set to 289.58–Φ_SA_ eV for calibration [33]. By looking up references, we found that the Φ_SA_ value of the MoS_2_ film is approximately 4.71 eV [7]. Thus, the C 1 s binding energy was set to 284.87 ± 0.21 eV in this work. The Z3 film was selected for fitting by a Gaussian–Lorentzian function, as shown in Figure 4b, the S 2p peaks at 162.2 eV and 163.4 eV correspond to the S 2p_3/2_ and S 2p_1/2_ spin-orbit components of MoS_2_, respectively. However, the observed S/Mo ratio deviates from the ideal stoichiometric value of 2:1. Additional peaks at 161.5 eV and 162.7 eV are identified as the S 2p_3/2_ and S 2p_1/2_ signals of MoS_2−x_, indicating the presence of sulfur vacancies [34]. The Mo 3d spectra are displayed in Figure 4c; the fitted Mo 3d peaks at 229.2 eV and 232.4 eV are attributed to Mo^4+^ in MoS_2_, corresponding to the Mo 3d_5/2_ and Mo 3d_3/2_ orbitals, respectively. The additional peaks at 228.6 eV and 231.2 eV are assigned to a doublet of Mo in MoS_2-x_. The other peak approximately at 227.0 eV is the S^2−^ state in MoS_2_ [34,35]. For Zr 3d spectra, as shown in Figure 4d, the peaks at 182.4 eV and 185.0 eV correspond to ZrO_2_, while the peaks at 181.4 eV and 183.9 eV are attributed to sub-stoichiometric zirconium oxides (ZrO_2−x_) [34]. With a further increase in the Zr target power, for Z4 film (Figure 4e), the Zr 3d spectrum has two additional peaks at around 180.1 eV and 182.7 eV, representing the metallic Zr, which is consistent with the findings reported by Li et al. [17]. In addition, no high energy peak such as 235.5 eV attributed to Mo-O bonds was found in the Mo 3d spectrum. This implies that Zr element is more likely to react with oxygen preferentially.

**Figure 5:**
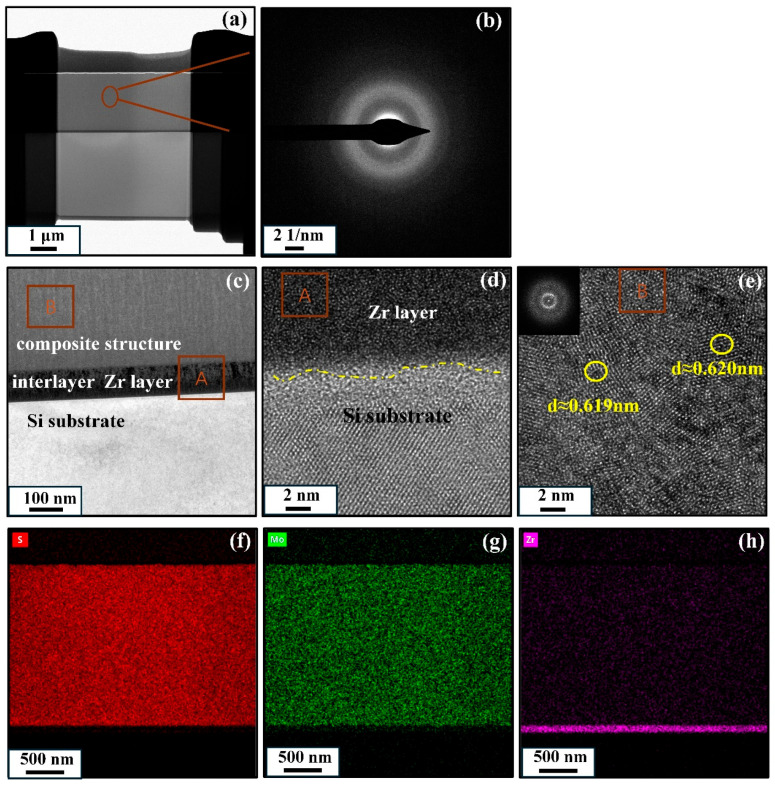
TEM images of Z3 film: (**a**) the cross-sectional image; (**b**) SAED pattern from the area marked as a circle in (**a**); (**c**) the enlarged film from (**a**); (**d**,**e**) the corresponding high-magnification image of marked section A and B on the film; and (**f**–**h**) the EDS mapping of the Z3 film.

In order to further investigate the microstructure of MoS_2_-Zr composite film, the typical Z3 composite film was selected for characterization. As shown in Figure 5a, the film presents a dense structure. The selected-area electron diffraction (SAED) pattern (see inset marked as circle in Figure 5a) in Figure 5b showed almost a continuous diffraction ring pattern, implying that the film is almost amorphous with few nanocrystals. Figure 5c is a partial enlarged view of Figure 5a, and it is clear that the composite film has double layer structure, which is composed of a Zr interlayer and a MoS_2_-Zr composite layer. The thickness of the Zr layer is around 100 nm. The areas in the bottom and middle are marked as A and B, respectively. A high-resolution image of region A, shown in Figure 5d, illustrates the interface between different layers of the film. The absence of a clear boundary between the Zr interlayer and the film above suggests strong interfacial bonding and good structural integrity across the multilayer architecture. The high-magnification image of region B in Figure 5e reveals a measured d spacing of approximately 0.620 nm, which is consistent with the (002) crystal plane of the MoS_2_ film. The d spacing of the (002) plane is slightly larger than that of bulk MoS_2_, which can be attributed to the disorder effects induced by Zr doping [36]. No Zr nanocrystals are detected, indicating that Zr primarily exists in the form of a solid solution within the MoS_2_ matrix. This is also consistent with previous XRD result. Based on the STEM mapping, as shown in Figure 5f–h, the Mo and S exhibit uniform and consistent distribution across the film, and although the Zr content is relatively low, it is also homogeneously distributed within the film layer.

**Figure 6:**
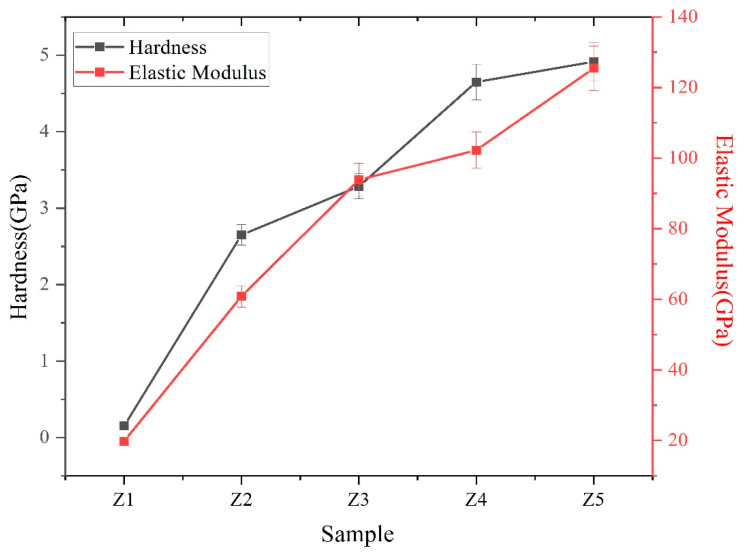
The hardness and elastic modulus of MoS_2_-Zr composite films with various Zr contents.

The hardness (H) and elastic modulus (E) of MoS_2_-Zr composite films are shown in Figure 6. With increasing Zr target power, both the hardness and elastic modulus of the films exhibit a gradual upward trend. The Z5 film achieves its maximum mechanical performance with a hardness of 4.9 GPa and an elastic modulus of 125.5 GPa. The enhancement in hardness and surface strength is mainly attributed to the solid solution strengthening effect and the microstructural densification caused by Zr incorporation. Interstitial Zr atoms introduce lattice distortion and hinder dislocation motion, while the denser structure with fewer intercolumnar voids improves load-bearing capacity and adhesion [37,38]. Additionally, Zr captures residual oxygen, suppressing Mo-O bond formation and further stabilizing the layered structure. These combined effects account for the observed hardness improvement.

**Figure 7:**
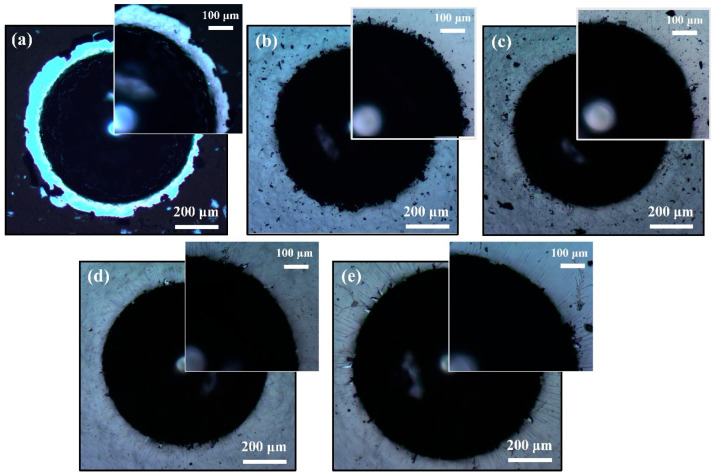
The indentation morphologies of MoS_2_-Zr composite films with various Zr contents: (**a**) Z1; (**b**) Z2; (**c**) Z3; (**d**) Z4; and (**e**) Z5 film.

Figure 7 presents a comparative analysis of the indentation morphologies of pure MoS_2_ films and MoS_2_-Zr composite films with varying Zr doping contents, observed under an optical microscope. The results reveal that the pure MoS_2_ film exhibits significant edge delamination, accompanied by the formation of numerous flake-like fragments, corresponding to an adhesion strength rating of HF6. When the Zr doping concentration increases to 3.60 at. % and 7.20 at. % (Z2 and Z3 film), the extent of interfacial delamination is markedly reduced, with the adhesion ratings improving to HF3. Notably, at a Zr content of 13.86 at. % (Z4 film), delamination is further suppressed, and the adhesion strength reaches HF2. However, when the Zr doping exceeds this critical value, the adhesion strength exhibits slight fluctuations, with ratings varying between HF2 and HF3. These experimental findings indicate that, compared with the pure MoS_2_ film, the incorporation of Zr can enhance the adhesion strength of the composite films by approximately 3–4 levels (from HF6 to HF2/HF3). This improvement is primarily attributed to the following mechanisms: (1) the increase in microstructural density of the composite films with higher Zr content; and (2) the simultaneous enhancement of material hardness, which effectively improves the films’ resistance to plastic deformation, thereby enhancing the interfacial adhesion performance.

**Figure 8:**
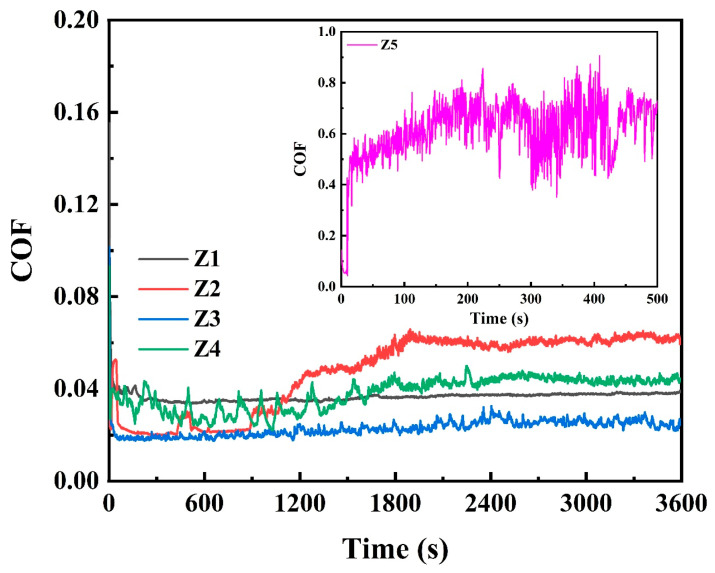
The friction coefficient curves of MoS_2_-Zr composite films under a vacuum condition.

Figure 8 shows the evolution of the friction coefficient curves for MoS_2_-Zr composite films in a vacuum environment, while Table 3 summarizes the corresponding average friction coefficients and wear rates for all films. The results indicate that with increasing Zr target power, the tribological performance of the films gradually improves, characterized by decreases in both friction coefficient and wear rate. Although the pure MoS_2_ film exhibits a relatively stable friction coefficient of approximately 0.036, it undergoes rapid wear-through and failure during sliding tests. This poor wear resistance is attributed to the low hardness and elastic modulus of the pure MoS_2_ film. As the Zr sputtering power increases, the friction behavior initially exhibits a slight rise followed by a pronounced decrease. Notably, the composite film with 7.20 at. % Zr (Z3 film) demonstrates the best tribological performance, with a stable friction coefficient of 0.022 and a minimum wear rate of 6.23 × 10^−8^ mm^3^/N·m. This enhanced performance can be attributed to two main factors. On the one hand, the XRD patterns suggest that moderate Zr incorporation is associated with a relative enhancement of the (002) orientation in the MoS_2_ matrix, which favors basal plane sliding under vacuum conditions. In contrast, the pure MoS_2_ film exhibits multiple orientations, including (002), (100), and (110), which may result in less efficient interlayer shear. On the other hand, increasing Zr content promotes microstructural densification, leading to improved hardness and elastic modulus, thereby enhancing load-bearing capacity and wear resistance. However, when the Zr sputtering power is further increased, the tribological performance deteriorates. At a Zr content of 19.03 at. % (Z5 film), the film rapidly fails at the initial stage of sliding. This degradation is likely related to excessive Zr incorporation, which disrupts the continuity of layered MoS_2_ domains and weakens the basal plane-dominated lubrication mechanism under vacuum.

**Figure 9:**
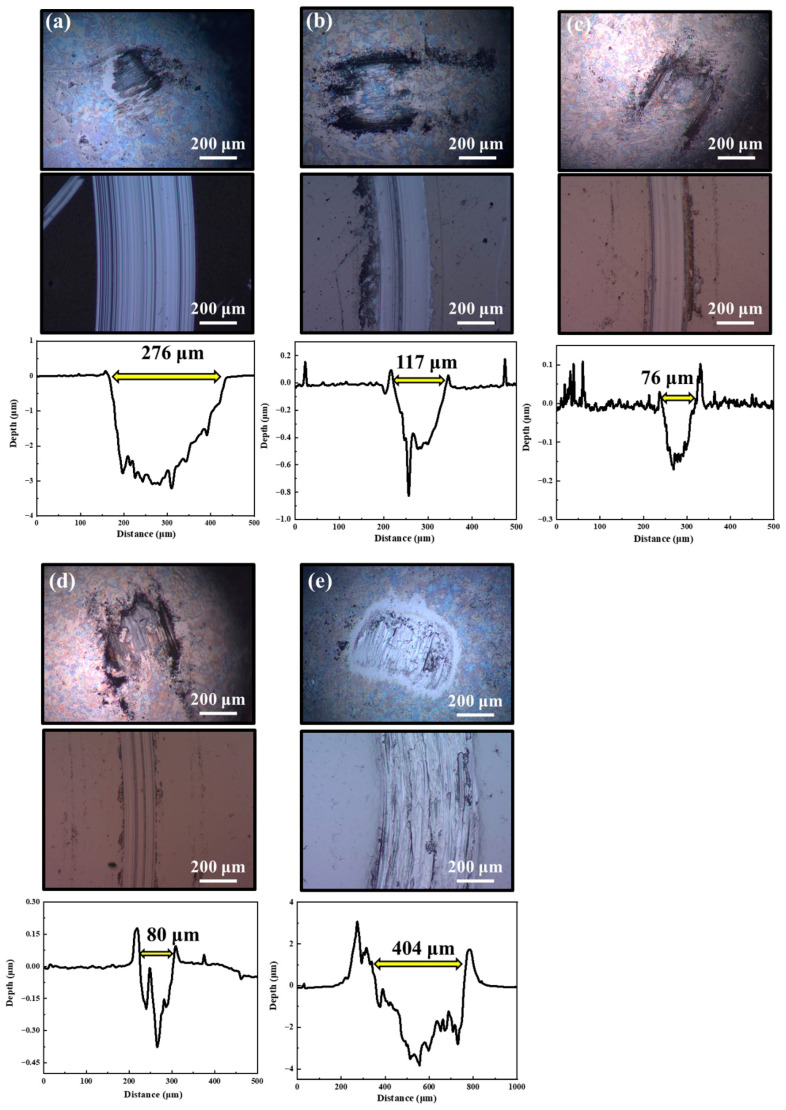
The morphologies of friction balls, wear tracks, and wear depths on the MoS_2_-Zr composite films under a vacuum condition: (**a**) Z1; (**b**) Z2; (**c**) Z3; (**d**) Z4; and (**e**) Z5 film.

Figure 9 presents the two-dimensional wear tracks and corresponding friction ball scar morphologies of MoS_2_-Zr composite films under a vacuum friction condition. Obviously, the pure MoS_2_ film exhibits a wear track width of ~276 µm and a maximum depth of ~3.2 µm, which exceeds the thickness of the film, indicating that the film has been worn through. For Z2 film, the wear track narrows to ~117 µm and shrinks to 0.85 µm depth. As for Z3 film, the film achieves its best wear resistance, with a track width of ~76 µm and only ~0.17 µm depth. For Z4 film, the values for wear track and depth is about 80 µm and 0.43 µm, respectively. For Z5 film, the wear depth of film increases to 3.8 µm, exceeding the film thickness. The depth of the wear track on films Z2–Z4 do not exceed the thickness of the films. A dark gray layer was observed in the central area of the friction ball scars, which is indicative of transfer layer formation during sliding, and obvious furrows were observed in the wear tracks of the films. These characteristics indicate that the friction process mainly involves two mechanisms: abrasive wear and adhesive wear. The wear track of Z5 film was found to be very rough due to the excessive metal adhesives on the surface of the counterpart ball; severe adhesive wear is the main wear mechanism. It can be concluded that as the Zr content increases, the tribological properties of the films gradually improve. When the Zr content reaches 7.20 at. %, the films have the best tribological properties. However, with the further increase in Zr content, the tribological properties of the film gradually deteriorate until it completely loses its lubricating function under vacuum conditions.

**Figure 10:**
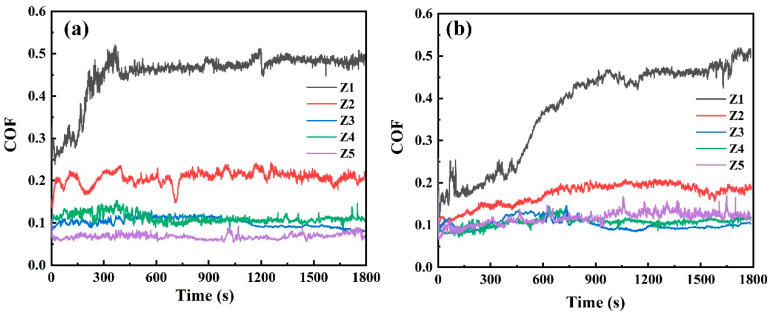
The friction coefficient curves of MoS_2_-Zr composite films: (**a**) atmospheric condition and (**b**) humid condition.

Figure 10 shows the variation of the friction coefficient of MoS_2_-Zr composite films deposited under different Zr sputtering powers in both ambient air and humid environments, while Table 4 summarizes the average friction coefficients and wear rates of these films. Overall, the MoS_2_-Zr composite films exhibit superior tribological performance compared with pure MoS_2_ film. As seen in Figure 10a,b, the pure MoS_2_ film fails rapidly within a short sliding duration under both ambient and humid conditions. With increasing Zr target power, the friction coefficients of the MoS_2_-Zr films decrease progressively during sliding. Among all films, the Z3 film, with a Zr content of approximately 7.20 at. %, shows the lowest friction coefficient and wear rate of 0.10 and 1.23 × 10^−6^ mm^3^/N·m in ambient air and 0.10 and 1.80 × 10^−6^ mm^3^/N·m in humid atmosphere, respectively. This improvement is attributed to two factors: first, the enhanced densification and increased hardness of the MoS_2_-Zr films with higher Zr incorporation; and second, the active oxidation surface of (100) in the MoS_2_-Zr composite film was effectively suppressed, both of which synergistically contribute to the excellent tribological performance of the MoS_2_-Zr film.

It is worth noting that, unlike in vacuum, the Z5 film exhibits the lowest friction coefficient in air. Under vacuum conditions, lubrication is primarily governed by basal plane sliding of MoS_2_, and excessive Zr disrupts layered continuity, thereby degrading performance. In contrast, under atmospheric conditions, lubrication is influenced not only by MoS_2_ shear but also by tribo-oxidation processes and the formation of adaptive surface layers. The presence of Zr facilitates the formation of stable zirconium oxide species during sliding, which may contribute to improved friction stability in air [39]. Consequently, films with higher Zr contents maintain reduced friction coefficients in atmospheric environments.

**Figure 11:**
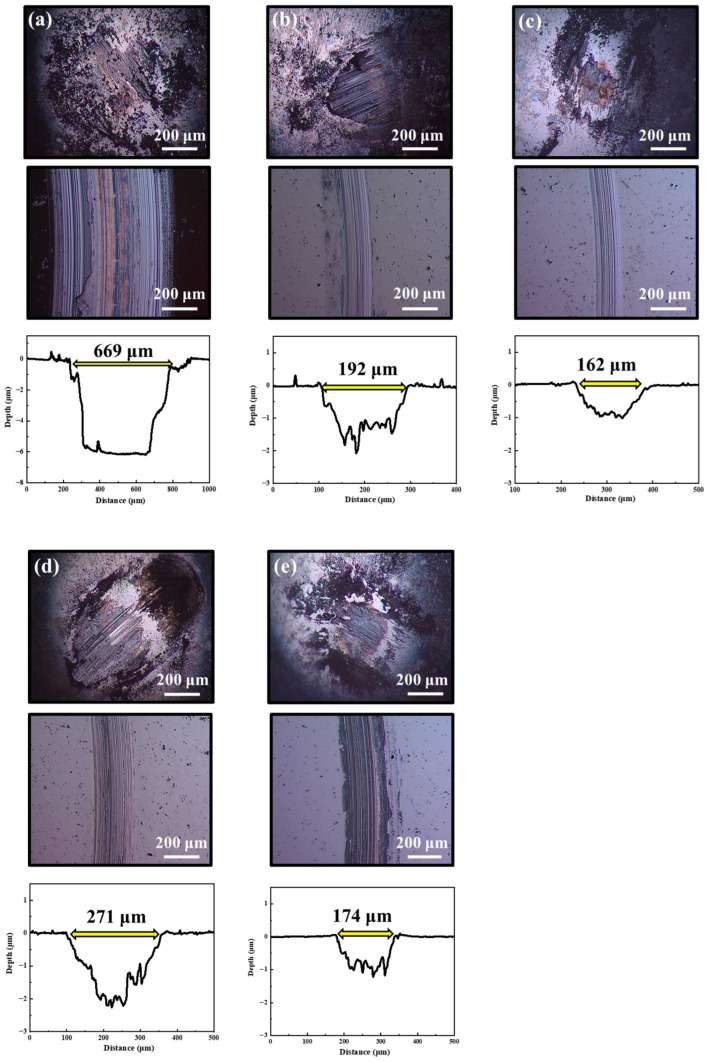
The morphologies of friction balls, wear tracks, and wear depths on the MoS_2_-Zr composite films under atmospheric conditions: (**a**) Z1; (**b**) Z2; (**c**) Z3; (**d**) Z4; and (**e**) Z5 film.

**Figure 12:**
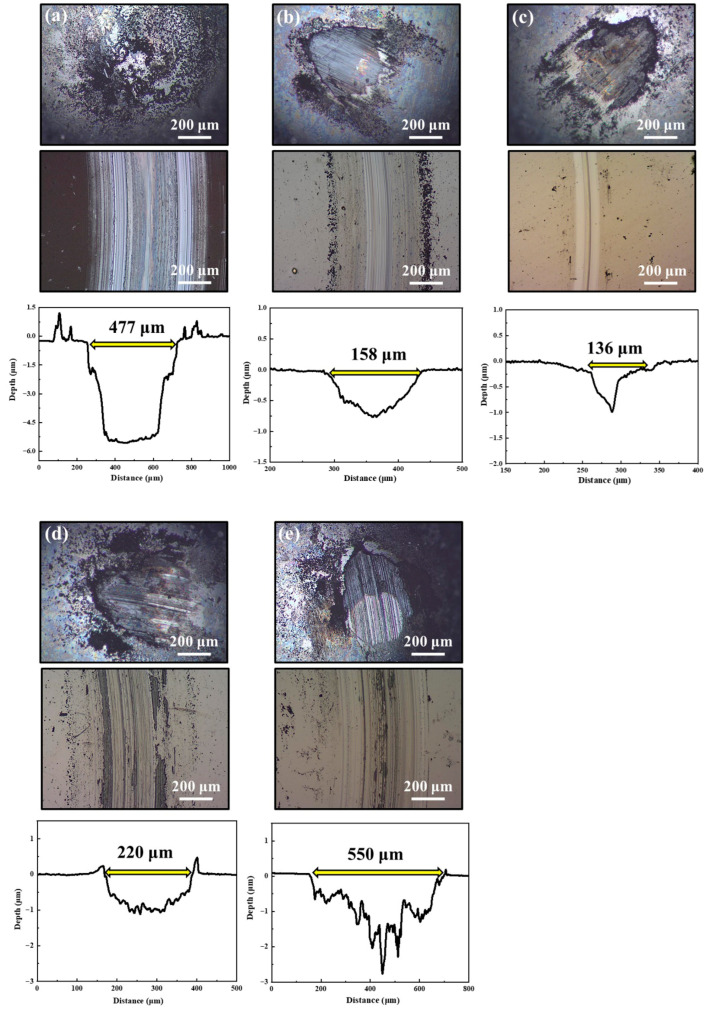
The morphologies of friction balls, wear tracks, and wear depths on the MoS_2_-Zr composite films under humid conditions: (**a**) Z1; (**b**) Z2; (**c**) Z3; (**d**) Z4; and (**e**) Z5 film.

Figure 11 and Figure 12 show the two-dimensional optical microscope morphologies of the wear tracks on MoS_2_-Zr composite films and the corresponding wear scars on the counterpart balls after sliding in atmospheric and humid environments. For the pure MoS_2_ films, the wear-track depths in both environments exceed the film thickness, indicating complete film failure. Prominent plowing grooves are observed on the worn surfaces, suggesting that abrasive wear is the dominant wear mechanism. In contrast, for the Z2–Z5 composite films, the wear track depths remain below the film thickness, and the plowing features are markedly shallower than that of pure MoS_2_. In addition, a gray layer is evident at the center of each ball scar, which suggests material transfer between the film and the counterpart during sliding. These observations suggest that the dominant wear mechanisms for the Z2–Z5 films involve a combination of abrasive wear and adhesive wear. Among them, the Z3 film exhibits the narrowest wear tracks and the lowest wear volume. However, starting from the Z4 film, both the width and depth of the wear tracks gradually increase with further Zr addition. This degradation in wear resistance is attributed to the excessive presence of metallic Zr in the films. Particularly in humid environments, the presence of water molecules changes the interfacial chemistry. Excessive Zr addition promotes partial oxidation and hydroxylation, generating Zr-O-H compounds rather than stable ZrO_2_. These hydrophilic phases can absorb moisture and disrupt the layered MoS_2_ structure, leading to higher shear resistance and debris generation during sliding, which exacerbates film wear. In summary, the tribological tests demonstrate that incorporating an optimal Zr content significantly enhances the friction and wear performance of MoS_2_-Zr composite films under all three alternating environments.

## 4. Conclusions

MoS_2_-Zr composite films were successfully fabricated by magnetron sputtering. The influence of Zr content on the composition, structure, and mechanical and tribological properties of films was systematically investigated. The Zr content could be precisely regulated by adjusting the target power. With increasing Zr doping, the structure of the film evolves from the loose columnar structure characteristic of pure MoS_2_ to a denser columnar structure in the MoS_2_-Zr composite films. At moderate Zr levels, the films show a strong preferential growth along the (002) crystallographic plane. However, with further Zr incorporation, the (002) diffraction peak gradually broadens. Combined XRD and TEM analyses reveal that the crystal structure gradually transforms into a dual-phase mixture of nanocrystals and amorphous regions. Both the hardness and elastic modulus of the films increase with rising Zr content, primarily due to solid solution strengthening and structural densification.

The tribological performance of the MoS_2_-Zr composite films is significantly enhanced compared with pure MoS_2_ film across vacuum, atmosphere, and humid environments. The friction coefficient and wear rate decreased initially with Zr addition, reaching optimal performance at 7.20 at. % Zr (Z3 film), which exhibited the lowest wear rates of 6.23 × 10^−8^ mm^3^/N·m in vacuum, 1.23 × 10^−6^ mm^3^/N·m in air, and 1.80 × 10^−6^ mm^3^/N·m in humid conditions. The improved performance is associated with microstructural densification, enhanced hardness, and modified surface interactions during sliding. Morphological observations of the counterpart surfaces suggest the formation of transfer layers, which may contribute to friction stabilization. The dominant wear mechanisms in the composite films are abrasive wear and adhesive wear. In conclusion, the properties of MoS_2_-based composite films could be tailored by optimizing the metal doping content, making it promising in applications as protective films in alternating environmental conditions.

## Figures and Tables

**Table 1:** The parameters for the deposition of MoS_2_-Zr composite films.

Parameters	Zr	MoS_2_-Zr
Base pressure (Pa)	5 × 10^−3^	5 × 10^−3^
Working pressure (Pa)	0.65	0.65
Bias voltage (V)	−50	−50
Rotation speed	3 r/min	3 r/min
Zr target power (W)	400	0/50/100/200/300
MoS_2_ target power (W)	0	300 + 300
Working temperature (°C)	120	120
Duty cycle	70%	70%
Deposition time (min)	10	90
Samples	/	Z1/Z2/Z3/Z4/Z5

**Table 2:** The composition of MoS_2_-Zr composite films.

Sample	Atomic Percentage/at. %			S/Mo
	Mo	S	Zr	
Z1	32.83	67.17	---	2.04
Z2	32.64	63.76	3.60	1.95
Z3	31.30	61.50	7.20	1.96
Z4	29.71	56.43	13.86	1.89
Z5	28.74	52.23	19.03	1.81

**Table 3:** The average friction coefficient (COF) and wear rate of MoS_2_-Zr composite films in a vacuum environment.

Sample	COF	Wear Rate (mm^3^/N·m)
Z1	0.036	Wear Out
Z2	0.047	3.37 ± 0.20 × 10^−7^
Z3	0.022	6.23 ± 0.12 × 10^−8^
Z4	0.038	1.08 ± 0.18 × 10^−7^
Z5	Wear Out	Wear Out

**Table 4:** The average friction coefficient (COF) and wear rate of MoS_2_-Zr composite films in air and humid environments.

Sample	COF (Air)	COF (Humid)	Wear Rate (mm^3^/N·m) (Air)	Wear Rate (mm^3^/N·m) (Humid)
Z1	Wear Out	Wear Out	Wear Out	Wear Out
Z2	0.20	0.17	2.75 ± 0.28 × 10^−6^	2.43 ± 0.34 × 10^−6^
Z3	0.10	0.10	1.23 ± 0.11 × 10^−6^	1.80 ± 0.10 × 10^−6^
Z4	0.11	0.11	7.11 ± 0.08 × 10^−6^	5.63 ± 0.15 × 10^−6^
Z5	0.07	0.12	3.87 ± 0.14 × 10^−6^	1.76 ± 0.18 × 10^−5^

## Data Availability

The original contributions presented in this study are included in the article. Further inquiries can be directed to the corresponding authors.
